# Efficacy and safety of an adsorbent and anti-oxidative vaginal gel on CIN1 and 2, on high-risk HPV, and on p16/Ki-67: a randomized controlled trial

**DOI:** 10.1007/s00404-020-05816-8

**Published:** 2020-11-20

**Authors:** Attila Louis Major, Vladimír Dvořák, Jana Schwarzová, Aleš Skřivánek, Tomáš Malík, Marek Pluta, Ivanna Mayboroda, Etienne Marc Grandjean

**Affiliations:** 1 Femina Gynecology Center, Rue Emile-Yung 1, 1205 Geneva, Switzerland; 2 Department of Obstetrics and Gynecology, Cantonal Hospital, University of Fribourg, Fribourg, Switzerland; 3 Centrum ambulantní Gynekologie a primární péče, s.r.o., Brno, Czech Republic; 4 Smetanova 388, 252 64 Velké Prilepy, Prague-West, Czech Republic; 5 G-CENTRUM Olomouc, s.r.o., Olomouc, Czech Republic; 6 Gyneko spol. s.r.o., Vsetin, Czech Republic; 7 Fakultní Nemocnice v Motole (University Hospital Motol), Onkogynekologická a kolposkopická Ambulance, Praha, Czech Republic; 8 Department of Obstetrics and Gynecology, University Hospital of Geneva, Geneva, Switzerland; 9 Phidalsa Pharma-Consultants, Petit-Lancy/Geneva, Switzerland; 10 Camara-Partners, Consultant, Nyon, Switzerland

**Keywords:** Cervical intraepithelial neoplasia, HPV, p16/Ki-67 dual staining, Non-surgical treatment, Silicon dioxide, Sodium selenite

## Abstract

**Purpose:**

The effect of SAM vaginal gel, a medical device containing adsorptive silicon dioxide and antioxidative sodium selenite and citric acid, on histologically-proven cervical intraepithelial neoplasia type 2 (CIN2) as well as p16 positive CIN1, and on the presence of the onco-marker p16 was investigated.

**Methods:**

216 women aged 25–60 years were randomized to either receive an intravaginal daily dose of SAM gel for three 28-day periods, or be followed-up without intervention. The primary endpoint was efficacy, defined as a combined histological and cytological regression. At baseline and after 3 months participants had: a guided biopsy including p16 immunohistochemical (IHC) staining, only if a lesion was visible at colposcopy; a cervical smear for cytology, high-risk human papillomavirus (hr-HPV) and a p16/Ki-67 test. At 6 months a further cytology and p16/Ki-67 test was performed.

**Results:**

Regression of CIN lesions was observed in 78 out of 108 patients (72.2%) in the SAM gel arm and in 27 out of 108 patients (25.0%) in the control arm. Similarly, the change in the p16/Ki-67 cytological test status was significantly in favor of the treatment arm. The prevalence of hr-HPV decreased significantly (*p* < 0.001) in the treatment arm, from 87.0% to 39.8%, while it slightly increased in the control arm, from 78.7% to 83.3%. At 6 months the cytological regression in the treatment group and the highly significant effect on p16/Ki-67 was still present.

**Conclusion:**

SAM vaginal gel enhances the regression of cervical lesions and clears hr-HPV and p16/Ki-67 in smears significantly, thus offering an active non-destructive management to prevent cervical cancer.

**Trial registration number:**

ISRCTN11009040, date of registration: 10/12/2019; https://doi.org/10.1186/ISRCTN11009040; retrospectively registered.

**Electronic supplementary material:**

The online version of this article (10.1007/s00404-020-05816-8) contains supplementary material, which is available to authorized users.

## Introduction

Cervical cancer is the main cause of oncologic death in many developing countries, whereas in developed countries it is rare and mainly observed in postmenopausal women. In developed countries, however, screening of cervical cancer is extremely costly and often results in hardly justifiable use of destructive methods on the cervix, inducing dyspareunia, cervical stenosis and premature delivery [1]. There is yet no single validated non-surgical therapeutic approach for mild to moderate CIN. Till today the attempts to efficiently treat human papilloma virus (HPV)-related low grade lesions with a non-destructive method have failed, either due to important adverse events (Imiquimod, Interferon) or due to an unsatisfactory response (green tea, metronidazole-containing gel, 5-fluorouracil (5-FU) vaginal cream) [2–4]. Even intralesional injection of interferon did not produce the expected effect [5]. Photodynamic therapy using a topical hexyl-aminolevulinic acid (ALA) cream followed by the application of a light-emitting device by the patient herself, had only a limited effect [6, 7]. Corrosive treatment of the cervical surface with topical 85% trichloroacetic acid in an outpatient setting showed promising results, but with not negligible vasovagal symptoms immediately after treatment [8]. Recently, an aqueous hydrocolloid vaginal gel was developed, containing highly dispersed silicon dioxide and DEFLAMIN® as active agents. Highly dispersed silicon dioxide is well established as a pharmacologically inert but adsorbent agent. The capacity of silicon dioxide to bind proteins, lipids, and lipoproteins non-specifically and thus to bind potential pathogenic agents results from the property of the charged surface structure [9–12]**.** DEFLAMIN® is a combination of sodium selenite and citric acid, according to a patented formula with utmost antioxidative properties [13].

DEFLAMIN® has already been applied successfully for treating topical irritations like herpes simplex, skin irritations after insect bites or sunburn, stomatitis aphthosa and parodontitis. The dosage of sodium selenite and citric acid corresponds to the concentrations of these components, used for the indications mentioned above [13]. Oxidative stress induced by infections and inflammation plays an important role in carcinogenesis and opens the door for new treatment options. Research suggests that oxidative stress is a key event for HPV DNA integration, which is an important step for malignant transformation of the cervical epithelium [14]. It was shown that oxidative damage of DNA is a multistep process increasing from CIN1 to CIN3 [15]. For this reason an early treatment of high risk CIN1 and of CIN2 with an anti-oxidative and adsorbent topical medical device may be a good option to prevent progression of CIN.

Recently a retrospective data analysis conducted by the Sigmund Freud University (SFU, Vienna) demonstrated that a high proportion of patients treated with the same vaginal gel, showed highly significant improvements of moderate pathological cervical smears within 16 weeks (70.7% vs 10.8%) [16]. These results suggested that the application of the vaginal gel might increase the spontaneous remission rate of abnormal cervical smears. However, the retrospective observational design of the study and the exclusively cytological findings are not considered sufficient. In order to improve the accuracy of screening and diagnosis, histopathological examinations, in addition with biomarkers, are required. A systematic review of hr-HPV screening alone or with cytology showed higher colposcopy consultations and a tendency for more destructive treatments, which may increase preterm deliveries [1, 17]. As a consequence, a number of onco-markers have gained relevance, among them especially IHC p16 for diagnosis and p16/Ki-67 dual staining for screening, which have been progressively recognized as prognostic factors and are currently recommended [18]. In hr-HPV positive women it was recently proposed that the cytological examination should be substituted by the p16/Ki-67 test, due to both a better sensitivity and a better specificity of the dual staining test compared to cytology [19, 20].

## Methods

The present investigation is a prospective, open, two-arm, controlled, multicenter trial comparing the efficacy of SAM gel with a non-treated control arm. The study was conducted at 3 gynecological centers in the Czech Republic (Brno/Vsetin, Olomouc, Prague). The clinical investigation comprised a 3-month treatment period and a follow-up duration of 6 months from treatment start. The treatment arm was subject to 3 × 28 day intravaginal application periods of the SAM gel containing 10.0 mg highly dispersed silicon dioxide, 24.8 mg citric acid, and 0.25 mg selenium per application (5 ml). 5 ml of the vaginal gel had to be applied daily deep inside the vagina using a single-use applicator.

The control arm patients underwent a no treatment intervention following the strategy of “wait and watch”, because in the current gynecological practice, there is no active treatment registered or recommended for the patients corresponding to the inclusion criteria.

Inclusion criteria were female patients at the age of 25–60 years; histological diagnosis of CIN1 and cytological Atypical Squamous Cells cannot exclude HSIL (ASC-H), Atypical Squamous Cells of Undetermined Significance (ASC-US), Low-grade Squamous Intraepithelial Lesion (LSIL) associated with a positive cytological p16 or histological p16 test [27]; or CIN2 or cytological High-grade Squamous Intraepithelial Lesion (HSIL); signed informed consent; a negative pregnancy test; a suitable method of contraception during the treatment period for women of childbearing age.

Exclusion criteria were oncological or immunological disease, chronic viral disease incl. hepatitis, immunosuppressive treatment, pregnancy or breastfeeding, known allergy to the gel or one of its components, colposcopy finding suspicious of invasive disease, simultaneous participation at another clinical trial, as well as, for CIN2 patients, unsatisfactory colposcopy (i.e. the transformation zone and/or the lesion is not fully visible) and for CIN1 patients, risk discrepancy with cytological finding (HSIL).

**Fig. 1:**
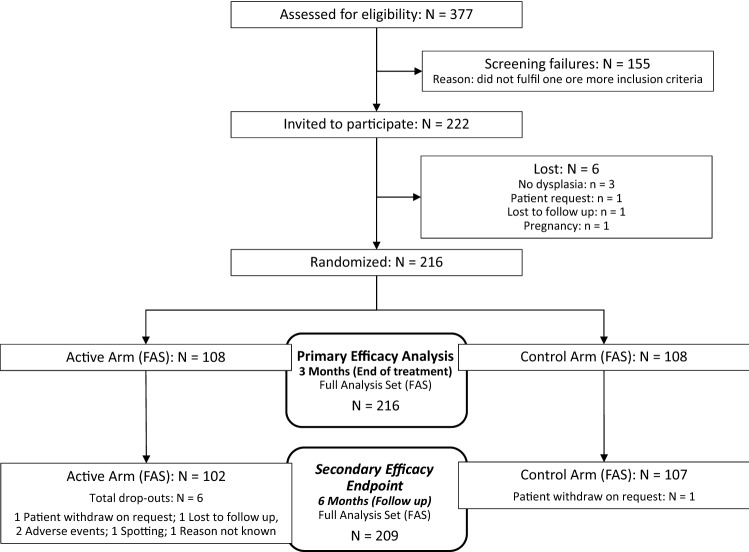
Flow diagram

377 patients were screened. 222 of them were invited to participate and in total, 216 patients were randomized (108 in each arm) between 9-May-2017 and 29-July 2018 (Fig. 1). Patients were block-randomized 1:1 to the SAM vaginal gel arm or "wait and watch" (control) arm. The investigational device was provided by the sponsor DEFLAMED International s.r.o., Prague, Czech Republic. SAM gel was allocated to the patients based on the randomization list.

Two independent histological experts in cervical histopathology were involved in the classification of the lesions and in the immuno-histochemical analysis. They were blinded in regards to the treatment arm and only the study number of the biopsy material was known. The examination of p16 by immunohistochemistry was interpreted according to the Lower Anogenital Squamous Terminology (LAST). To select CIN1 with higher risk of progression, only CIN1 p16 positive patients were included in the study [21–24].

Conventional cervical smears were used for initial cytology examination and immunocytochemistry tests. The smears were under special care transported to the laboratory (AeskuLab Patologie, k.s., Prague).

Cytological samples were submitted to the usual screening analysis, stained according to Papanicolaou and evaluated in accordance to the Bethesda classification. In addition, at the same time, a second test was used to perform immunocytochemistry dual biomarker technology CINtec® Plus Cytology, Roche.

Material from cervical smear was also taken for determination of hr-HPV status. Roche Cell Collection Medium was used for transport and cell preservation. Cobas® 4800 HPV Test (Roche Diagnostics, GmbH, Mannheim, Germany) were performed to identify 14 genotypes of hr-HPV DNA, with separate genotyping of 16 and 18 hr-HPV and the group of others (31, 33, 35, 39, 45, 51, 52, 56, 58, 59, 66, 68).

The primary endpoint of this trial was the regression rate comparison after three months of using SAM vaginal gel in the active arm and “watch and wait” patient control arm. The regression rate was defined as the combined endpoint of cytology and histology. Success was regarded as either cytological regression; defined as an initial ASC-US, LSIL, ASC-H or HSIL lesion which disappeared or changed to lower level (e.g. LSIL to ASC-US etc.) after treatment OR histological regression; defined as an initial CIN1 lesion which disappeared after treatment, or as an initial CIN2 lesion being replaced by CIN1 lesion or which disappeared after treatment, respectively.

The secondary endpoint was a regression or remission of cytopathological findings after 3 months treatment (active arm) or “watch and wait” (control arm) and a further 3 months follow up without treatment in both arms (visit 4, 6 months). This was analyzed by respecting the following order of decreasing risk of development of squamous cell carcinomas according to Bethesda: HSIL, ASC-H, LSIL, Atypical Glandular Cells (AGC), ASC-US. The other two endpoints were: cytological change in p16/Ki-67 (CINtec® Plus test) after 3 and 6 months; and the clearance of hr-HPV strains scored at 3 months [25].

Safety was assessed by adverse events and measurement of serum selenium levels. Blood sampling for selenium analysis was done at visit 1 and at visit 3. 5 ml of blood was collected. Serum selenium (reference interval: 0.71–1.83 µmol) was assessed by using atomic absorption spectrometry (AAS) with electrothermal atomization (ETA-AAS).

For final analysis, a sample of *N* = 100 evaluable subjects per arm (total *N* = 200) was originally planned to provide an overall cumulative trial power of 88% to demonstrate superiority of SAM vaginal gel to “wait and watch”. To this, 22 subjects were added to account for an expected dropout of patients of 10% during the follow-up period. Power computations were based on comparison of proportions using normal approximation with interim and final Alphas (0.0059 and 0.0516*,* respectively) determined by the O’Brien-Fleming method, with overall two-sided Alpha = 0.05. A total of *N* = 222 subjects was planned to participate in this trial.

All statistical computations and randomizations were done in Stata® 13 (Stat Corp Ltd., USA). The effects of noncompliance, dropouts, and possible covariates such as age were planned to be assessed to determine the impact on the general applicability of results from this study. Overall Type I Error was planned to be controlled at 0.05 for the primary analyses (including interim). Alpha for significance for each secondary analysis was 0.05; secondary analyses were considered of exploratory character and could therefore not control an overall Type I Error.

Additionally, the effect of age on the primary endpoint was tested using a logistic regression model adjusted for age and interaction term age × medication.

## Ethical approval

The study was approved by the Multicentre Ethics Committee (February, 2017) and the Local Ethics Committee (October, 2017).

The study was registered retrospectively on 10/12/2019 in the ISRCTN registry with the ID ISRCTN11009040, 10.1186/ISRCTN11009040.

## Results

The primary hypothesis was tested in the Full Analysis Set (FAS) i.e. Intention-to-Treat population (ITT) comprising 216 patients (Fig. 1). For the FAS at the 4th visit, 1 patient was excluded in the control arm and 6 in the active arm. The number of excluded patients between both arms was not significant (Fisher´s two-tailed exact test; *p* = 0.280).

Baseline characteristics are presented in Table 1. Several demographic data differences could be seen between the treatment and control arm. First, the distribution of treatment allocation between individual centers in the FAS population was uneven (Pearson’s chi-squared test; *p* < 0.001). This was caused by screening failures in the centers 1 and 2 and almost 1-year delay in study initiation in the center 3. The investigators had no influence on the distribution of the patients to the study arms. Stratification of the patients according CIN grade was not defined in the protocol. For this reason the distribution between CIN1 and CIN2 patients in the active and the control arm is uneven.

**Table 1:** Baseline characteristics

FAS population		Active arm (*n* = 108)		Control arm (*n* = 108)		*P*
Age (years) Mean ± SD		33	± 6.7	35.5	± 8.6	0.037^**+**^
Rel. gynecological history		5	4.6%	17	15.7%	0.012^**x**^
Smoking		33	30.6%	32	29.6%	1.000^x^
HPV vaccination		13	12.0%	15	13.9%	0.840^x^
Histology#	CIN1	56	51.9%	91	84.3%	< 0.001^**X**^
	CIN2	52	48.1%	17	15.7%	
	Total	108	100.0%	108	100.0%	
Cytology	NILM	6	5.60%	1	0.9%	0.129*
	ASC-US	22	20.4%	25	23.1%	
	AGC	1	0.9%	1	0.9%	
	LSIL	59	54.6%	59	54.6%	
	ASC-H	9	8.3%	17	15.7%	
	HSIL	11	10.2%	5	4.6%	
	Total	108	100.0%	108	100.0%	
High-risk HPV^S^	Yes	94	87.0%	85	78.7%	0.148^x^
	No	14	13.0%	23	21.3%	
	Total	108	100.0%	108	100.0%	
CINtec® Plus p16/Ki-67^SS^	CIN1	40/56	71.4%	87/91	95.6%	< 0.001^**x**^
	CIN2	37/52	71.2%	12/17	70.6%	
	Total	77/108	71.3%	99/108	91.7%	
IHC p16^SSS^	CIN1	50/56	89.3%	49/91	53.8%	< 0.001*
	CIN2	49/52	94.2%	17/17	100.0%	
	Total	99/108	91.7%	66/108	61.1%	
High-Risk HPV	CIN1	46/56	82.1%	69/91	75.8%	n.a.
	CIN2	48/52	92.3%	16/17	94.1%	
	Total	94/108	87.0%	85/108	78.7%	

Values given as mean, ± standard deviation, %; Statistical analysis by:

^+^ Wilcoxon rank-sum test

^X^ Fisher´s two-tailed exact test

*Pearson’s chi-squared test

^S^According to HPV Cobas® 4800 test

^SS^According to CINtec ®Plus Roche (p16/Ki-67)

^SSS^ According to CINtec® Roche (p16) Histology-Test

^#^CIN1 p16 positive (IHC or CINtec® Plus)

*n.a*. not analyzed

Furthermore, the patients in the control arm were slightly, but statistically significant, older. The effect of age on the primary endpoint was tested using a logistic regression model including age and interaction term of age with treatment. The entire model is significant with a *p*-value < 0.001 and a pseudo determination coefficient (*R*^2^) of 0.140, meaning that age was not responsible for overall regression rate and that the treatment is most likely to be effective regardless of the patient’s age.

In addition, relevant gynecological history (conservative surgeries of the uterus and surgeries for adnexal diseases) was significantly more frequently reported in the control arm and there were significantly more IHC p16 positive patients in the active arm at baseline. Biopsies also revealed that CIN2 was significantly more frequent in the active arm (52/108 patients) vs. the control arm (17/108 patients). Conversely, the control arm had a slightly higher rate (91.7%) of positive cytological p16/Ki-67 compared to the active arm (71.3%). The difference is due to the significantly higher CIN1 prevalence in the control arm (84.3%) in contrast to the active arm (51.9%). Patients with CIN1 were more often included in the study due to a positive cytological p16/Ki-67 test in the control arm (46.2%) compared to the active arm (10.7%) which was not confirmed by IHC p16 on the contrary. For all other baseline characteristics, the two arms were comparable (Table 1).

**Fig. 2:**
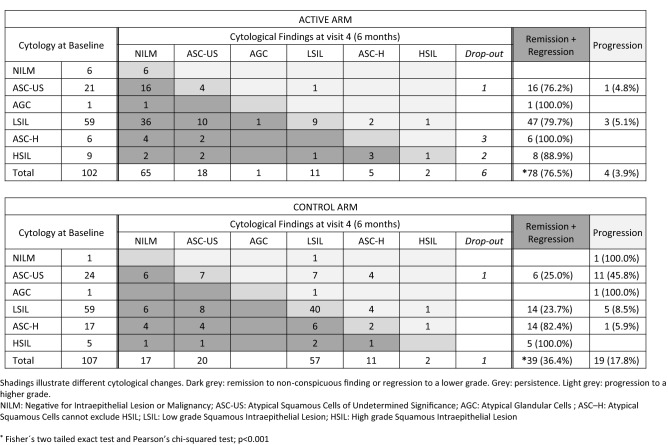
Difference in cytological findings after 6 months in the active and control arms

After 3 months of treatment (3rd visit), the overall histological and cytological regression using FAS population (primary endpoint) was significantly higher (*p* < 0.001) in the active arm (78/108 patients or 72.2%) than in the control arm (27/108 patients or 25.0%) (Table 2). The null hypothesis of no difference in regression rate can thus be rejected. At the same time-point, the purely cytological evolution was separately analyzed, as a secondary endpoint. 77 patients (71.3%) in the active arm and 27 patients (25.0%) in the control arm experienced cytological remission or regression (*p* < 0.001). This difference in efficiency was still present at 6 months. This effect at 6 months, however, was mostly due to resolving low grade cytological findings (ASC-US and LSIL). Cytological regression was observed in 78/102 patients (76.5%) in the active arm and 39/107 patients (36.4%) in the control arm (Fig. 2). The difference in cytological regression rate between arms was significant when analyzed as dichotomous yes/no (Fisher´s two-tailed exact test; *p* < 0.001), and also when analyzed as remission, regression, persistence and progression (Pearson’s chi-squared test; *p* < 0.001). The fate of any individual cytological finding is shown on Fig. 2 for the six months results. For example out of 59 LSIL findings at baseline in the active arm 36 patients (61.0%) became NILM, 10 patients (16.9%) became ASC-US, 1 patient (1.7%) became AGC, 9 patients (15.3%) remained LSIL, 2 patients (3.9%) progressed to ASC-H and 1 patient (1.7%) to HSIL. As a comparison out of 59 patients with LSIL at baseline in the control group, 6 patients (10.2%) became NILM, 8 patients (13.6%) became ASC-US and 40 patients (67.8%) remained LSIL, while 4 patients (6.8%) progressed to ASC-H and 1 patient (1.7%) to HSIL. Out of the 21 ASC-US findings in the active arm 16 patients (76.2%) improved to Negative for Intraepithelial Lesion or Malignancy (NILM), whereas in the control arm 6 patients (25.0%) showed remission to a non-conspicuous finding (Fig. 2).

**Table 2:** Primary endpoint: Regression rate of the combination of histological or cytological findings of CIN2 and CIN1 p16 positive after 3 months treatment with SAM gel among patients by SAM gel and control arm

Regression (primary endpoint)	Active arm			Control arm			*p* between arms*
Visit 1—Visit 3	Cytology	Histology	Cyto or Histo	Cytology	Histology	Cyto or Histo	
Present (success)	77 (71.3%)	19 (86.4%)	78 (72.2%)	27 (25.0%)	2 (20.0%)	27 (25.0%)	*P* < 0.001
Absent (failure)	31 (28.7%)	3 (13.6%)	30 (27.8%)	81 (75.0%)	8 (80.0%)	81 (75.0%)	
Total	108 (100.0%)	22 (100.0%)	108 (100.0%)	108 (100.0%)	10 (100.0%)	108 (100.0%)	

^*^Pearson’s chi-squared test/two-sample proportional test

The CINtec® Plus test showed, 13 (16.9%) out of 77 patients initially positive in the active arm remained positive at the end of treatment, while 89 (82.4%) became negative (Supplement 2). None of the 31 initially negative patients became positive at 3 months, while one of them missed the 3 months test. In the control arm, out of 99 patients initially positive, 81 (75.0%) remained positive, while 26 (24.1%) became negative at 3 months (*p* < 0.001). Two out of 8 initially negative patients had a positive test at 3 months. The difference in CINtec Plus® test between the arms (76.6% vs 20.2%) was significant in favor of the treatment group (Fisher´s two-tailed exact test; *p* < 0.001).

At 6 months the CINtec Plus® results were comparable to the 3 months data (Supplement 2). A detailed analysis at 6 months, i.e. 3 months after the end of treatment, showed that 60 (83.3%) out of 72 initially CINtec Plus® test positive patients were negative in the active arm, whereas in the control arm 21 (21.4%) out of 98 initially positive patients became negative. The difference of CINtec® Plus test results between the arms at 6 months was significant (Fisher´s two-tailed exact test; *p* < 0.001).

Regarding hr-HPV, 94 (87.0%) out of 108 patients were tested positive for hr-HPV in the active arm. After 3 months treatment, 43 patients were hr-HPV positive. Clearance had occurred in 54.3% of patients. No single patient was newly infected by hr-HPV. In the control arm, 85 (78.7%) out of 108 patients were positive for hr-HPV. At 3 months, 90 patients in the control arm were hr-HPV positive and 9 patients (10.6%) were cleared from hr-HPV, whereas 14 patients were newly infected (Supplement 2). While the number of patients with hr-HPV was comparable at baseline, there were fewer patients at the 3rd visit at high risk in the active arm (39.8%) compared to the control arm (83.3%). Thus, the difference in hr-HPV prevalence between arms was significant (Fisher´s two-tailed exact test; *p* < 0.001).

Considering the patients hr-HPV negative at baseline, the number of new positivity for hr-HPV at 3 months was 0 out of 14 cases in the active arm, and 14 out of 23 in the control arm.

During the full period of the study six drop outs were recorded in the active group and one in the control group. The total drop outs occurred after the 3 months treatment period with SAM vaginal gel during the 3 months follow up. One patient withdrew voluntarily and one dropped out for an unknown reason. Two patients decided not to enter the 3 months follow up period because of adverse events reported during the three months treatment period. In the control group there was one single voluntary withdrawal (Fig. 1).

During the treatment period 17 patients reported 42 adverse events (AE) in the active arm and 1 patient reported 1 AE in the control arm. Out of the recorded AE, 12, reported by 4 patients, were assessed to have an imputability being possible/not known, probable or causal (Table 3). Most AE were local (vaginal itching/burning, vaginal bloody discharge, increased vaginal bleeding, vaginal mycosis or herpes) as well as slight abdominal pain or cramps. None of them required the termination of device application. No serious possibly device-related adverse events occurred.

**Table 3:** Adverse events among patients by SAM gel and control arm

Active Arm				
Causality with the device	Severity	Patient ID	AE description	Number of entries
Not related	MILD	88, 146, 5	Abdominal pain	6
		272	Borreliosis after the Tick Bite	1
		88	Headache	1
		151	Heartburn	1
		146	Mild kidney problems	1
		88	Muscle pain	1
		76, 144	Nasopharyngitis	3
		88	Ovarian pain	1
		151	Swelling of the lower limbs	1
	Moderate	128	Appendectomy, phlegmone	1
		235	Blocked cervical spin	1
		146, 235	Headache	4
		85, 88	High fever	2
		151, 235	Worsening of atopic eczema, perioral dermatis	2
	Severe	145	Cystitis	1
		197*	Vaginal mycosis	1
Unlikely	Moderate	244	Herpes	1
		152	Tonsilitis	1
Total not related or unlikely	Number of patients	14	Total entries of AE	30
Possible/not known	Mild	26, 67	Cramps and tingling in the lower abdomen	4
		26, 62	Mild burning of the vagina	1
	Moderate	67	Abdominal pain	2
		67	Weak menstrual bleeding	1
		197*	Vaginal mycosis	1
	Severe	67	Severe menstrual bleeding	1
Probable	Moderate	62	Burning of the vagina	1
		62	Bloody discharge	1
Total possible/not known or probable	Number of patients	4	Total entries of AE	12
Total	Number of patients	17	Total entries of AE	42
*Control arm*				
Not related	Moderate	124	Tonsilitis	1

*Patient with ID 197 had 2 entries for vaginal mycosis with different allocation by the doctor to causality with the device

Serum selenium measurement at study start and at the 3rd visit (active arm only) confirmed that there is no systemic absorption of selenium.

## Discussion

This prospective, comparative, open, two-arm, controlled trial demonstrated that the medical device SAM vaginal gel is effective in the treatment of p16 positive CIN1 and of CIN2. SAM gel administration led to a significant treatment success shown by the increase of the regression rate of precancerous disease, 72.2% in the SAM gel arm vs 25.0% in the control arm. The significant decrease of hr-HPV (from 94 to 43 hr-HPV in the active arm) and CINtec® Plus tests (from 77 to 13 positive tests) compared to the control arm (watch and wait) were in line with the histo- and cyto-pathological observations. 3 months after the end of the SAM gel treatment its effect on regression (76.5% in active arm vs 36.4% in control arm) and on progression (3.9% in active arm vs 17.8% in control arm) could still be observed. The detailed analysis of the individual cytological findings at 6 months, as shown in Fig. 2, support a prolonged effect 3 months after completion of the gel application. The effect was more prominent in the low risk categories (ASC-US and LSIL) than in the high risk categories (ASC-H and HSIL).

To our knowledge it is the first time that a non-destructive local application on the vagina and cervix was investigated with promising results in a randomized study to treat p16 positive CIN1. It is also the first time that p16/Ki-67 were used as biomarkers of oncogenic hr-HPV infection in such a study. The p16 test used in the inclusion criteria of the study enabled to select CIN1 lesions which have a higher risk of progression [18]. CIN1 is the most frequent type of histopathology findings in biopsies of precancerous disease. It has a 12–16% progression rate to more advanced precancerous disease and 1% of CIN1 will develop to invasive cervical cancer. The goal is to select patients with high oncogenic risk and by its topical treatment with SAM gel to prevent overtreatment in the future [25]. Instead of the watch and wait strategy described in many guidelines, a gentle local application with the medical device SAM gel is proposed in this study.

In the Squamous Terminology project (LAST) LSIL/CIN1 contains two different categories: the benign CIN1 condyloma lesions with cytopathic effect of the virus and associated with low risk HPV; as well as the flat CIN1 lesions with dysplastic character and presence of high risk HPV in 80% of these lesions [26]. These flat CIN1 lesions have a progression rate to CIN 3 in 12%, a persistence in 32% and a regression in 57%. Around 50% of CIN1 present a positive block staining of the lower 1/3 of the epithelium with p16; and these are the lesions associated with hr-HPV [27]. In the novel terminology the p16 staining pattern is a criterion to upgrade CIN2 to CIN3. Only p16 positive CIN2 are considered as indication for a treatment such as conization or cryotherapy. The rationale behind not treating CIN1 surgically is that a big part of CIN1 will spontaneously regress. Therefore it is proposed to wait the evolution of CIN1 into CIN2 or CIN3 before initiating a treatment. The aim is to prevent unnecessary conizations or other destructive methods, since they are associated with preterm delivery, infertility, dyspareunia, psychological discomfort for the patient and additional costs for the health system. It has to be mentioned that the uterine cervix was underestimated in fertility in the past and that it is important not only to preserve the cervical length but also the production and function of the mucus by cervical glands [28].

A non-destructive method to treat precancerous disease in an early stage by its roots is therefore very welcome. It is important however to select cases of CIN1 with higher risk of progression. The tumor suppressor protein p16 affords this property. Whereas the data for treating p16 CIN1 lesions are not convincing enough to treat the cervix by destruction, they are strong enough to select CIN1 lesions for non-destructive treatments and as a consequence this should decrease conisations and unnecessary destruction of lesions which may have progressed. Sidhu et al. used 5-FU, a chemotherapeutical drug, topically with a new vaginal delivery system in a double blind randomized controlled trial for CIN1 and CIN2 lesions [29]. However the results were disappointing. The reason is probably due to the single vaginal application of 5-FU. On the other hand Rahangdale achieved with 8 applications of 2 g of topical 5% 5-FU cream (Efudex; Valeant Pharmaceuticals International, Quebec, Canada) during 4 months significantly improved regression rates for CIN2; 93% in the 5-FU group compared to 56% in the observational group [30]. To mention is that 5-FU may induce the side effect vaginal adenosis after topical 5-FU treatments of CIN1 [31]. CIN1 and CIN2, like in our study, were treated topically by Ashrafian in a randomized controlled trial, with 3,3′-diindolylmethane (DIM), a stable metabolite of Indole-3-carbinol (I3C). Due to the fact that no information is given concerning the distribution of CIN1 and CIN2, the groups are not comparable. Because of this lack of important information in this study a statement of the treatment efficacy is difficult [32]. Two other randomized controlled trials, however only for CIN2 and more ( +), showed significant treatment results. In one study 3 applications of the virus replication inhibiting Cidofovir gel in a cervical cap were used before conisation and in the other the immunomodulator Imiquimod with self-applied vaginal suppositories was used for a duration of 4 months [33, 34]. Because of local and systemic side effects Imiquimod is inappropriate for the treatment of CIN1 [35]. A new trial with Imiquimod for CIN2 + had to be stopped due to the lack of appropriate patients to be included in the study, because many preferred either the Large Loop Excision of the Transformation Zone (LLETZ) procedure or the Imiquimod treatment [36].

The results of the present trial provide important information. It confirms the hypothesis, that the application of the medical device SAM vaginal gel is associated with an overall histological and cytological regression. Furthermore HPV clearance was higher in the treatment arm than in the control arm. Nevertheless the present study is endowed with a few limitations, first of all the uneven distribution of some patients’ characteristics between treatment and control group.

However, it can be seen that patients in the active arm were more advanced in precancerous disease at baseline since it encompassed a higher number of CIN2 IHC p16 positive findings. Therefore, this difference can not hamper the results in favor of the treatment arm. Although patients in the control group are slightly, but significantly older than in the active group, this difference (33.0 versus 35.5 years) has little clinical impact. A further limitation of the study is, that no biopsy was performed at 3 months in patients with normal or unchanged colposcopy for ethical considerations. Another limitation is that the study was not placebo controlled, but the “watch and wait” control arm represents the state-of-the-art attitude.

This randomized study demonstrated that the medical device SAM vaginal gel is effective for enhancing the regression of cervical lesions and preventing their progression. The significant change in p16/Ki-67 shows that SAM gel is influencing oncogenic progress substantially, indicating that the vaginal gel is a potential therapy regimen for patients with HPV infected cervical lesions. Although such data have not been collected at longer term, the present results strongly suggest that the alteration of the vaginal milieu by the SAM gel during 3 months treatment is capable of reversing the oncogenic activity of hr-HPVs. One potential explanation could be the alteration of the vaginal microbiome influencing hr-HPV positivity and CIN prevalence [37]. 13% of patients (*n* = 14) in the control arm were newly diagnosed with hr-HPV-infection within 3 months. One part may be due to the rate of false negative results at the recruitment and of false positive results in the follow up, which is in the range of published data, the other part to new infections.

The SAM gel application proved to be quite safe, since only 4 patients out of 108 reported on possibly gel-related AE, all of which were local and slight. The main benefit of the vaginal gel is its simple non-destructive application during the time of “watch and wait” where no other treatment options are available. In the conditions of our study, this gel appeared to be an effective and safe therapy. The patient can apply the product herself and no clinic visit is necessary for application of the vaginal gel. Further studies with longer treatment and longer follow up are needed to evaluate the effect of SAM gel more accurately on the prevention of cervical cancer development.

## Electronic supplementary material

Below is the link to the electronic supplementary material.

Supplementary file1 (DOCX 22 kb)

Supplementary file2 (DOCX 27 kb)

## Data Availability

Clinical trial data is available to regulators, researchers, and trial participants upon request.
