# Supplementary material for: Efficacy and safety of an adsorbent and anti-oxidative vaginal gel on CIN1 and 2, on high-risk HPV, and on p16/Ki-67: a randomized controlled trial
Source: Arch Gynecol Obstet. 2020 Nov 20;303(2):501–11. doi: 10.1007/s00404-020-05816-8 (PMC7858556; doi:10.1007/s00404-020-05816-8)
Supplement: Supplementary file 1 — Supplementary file1 (DOCX 22 kb) [file 404_2020_5816_MOESM1_ESM.docx]

**Title:** Efficacy and safety of an adsorbent and anti-oxidative vaginal gel on CIN 1 and 2, on high-risk HPV, and on p16/Ki-67: a randomized controlled trial.

**Journal name:** Archives of Gynecology and Obstetrics

**Author names**

Attila Louis Major, MD PhD^1,2^, Vladimír Dvořák, MD PhD^3^, Jana Schwarzova, MD^4^, Ales Skrivanek, MD PhD^5^, Tomáš Malík, MD^6^, Marek Pluta, MD PhD^7^, Ivanna Mayboroda, MD^1,2^ , Etienne Marc Grandjean, MD^8^

Affiliation and e-mail address of the corresponding author:

1 Femina Gynaecology Centre, Geneva, Switzerland

2 Cantonal Hospital, University of Fribourg, Switzerland

**Corresponding author**

Prof. A.L. Major, Femina Gynaecology Centre, Rue Emile-Yung 1, 1205 Geneva, Switzerland, Tel ++41 22 347 62 22

E-Mail: [majorattila@outlook.fr](mailto:majorattila@outlook.fr)

Supplement 1: Schedule of Study Procedures

| **Visits**  **Day (D) / Month (Mo)** | **V1**  **D 7** | **V 2**  **D 28** | **V 3**  **D 84** | **V 4**  **Mo 6** |
| --- | --- | --- | --- | --- |
| Cervical smear | X |  | X | X |
| CINtec Plus (p16, Ki-67) | X |  | X | X |
| HPV status | X |  | X |  |
| Colposcopy ± Colpophotography | X |  | X | X |
| Histology & IHC p16 | X |  | X **^1^** |  |
| Blood sampling (Selenium) | X |  | X |  |
| Patient diary & adverse events (AE) |  | X | X | X**^2^** |
| ^1^ **performed only on a visible lesion**  ^2^ **Adverse Events Control only** | | | | |
