# Supplementary material for: Efficacy and safety of an adsorbent and anti-oxidative vaginal gel on CIN1 and 2, on high-risk HPV, and on p16/Ki-67: a randomized controlled trial
Source: Arch Gynecol Obstet. 2020 Nov 20;303(2):501–11. doi: 10.1007/s00404-020-05816-8 (PMC7858556; doi:10.1007/s00404-020-05816-8)
Supplement: Supplementary file 2 — Supplementary file2 (DOCX 27 kb) [file 404_2020_5816_MOESM2_ESM.docx]

**Title:** Efficacy and safety of an adsorbent and anti-oxidative vaginal gel on CIN 1 and 2, on high-risk HPV, and on p16/Ki-67: a randomized controlled trial.

**Journal name:** Archives of Gynecology and Obstetrics

**Author names**

Attila Louis Major, MD PhD^1,2^, Vladimír Dvořák, MD PhD^3^, Jana Schwarzova, MD^4^, Ales Skrivanek, MD PhD^5^, Tomáš Malík, MD^6^, Marek Pluta, MD PhD^7^, Ivanna Mayboroda, MD^8^ , Etienne Marc Grandjean, MD^9,10^

Affiliation and e-mail address of the corresponding author:

^1^ Femina Gynaecology Centre, Geneva, Switzerland

^2^ Cantonal Hospital, Department of Obstetrics & Gynecology, University of Fribourg, Switzerland

**Corresponding author**

Prof. A.L. Major, Femina Gynaecology Centre, Rue Emile-Yung 1, 1205 Geneva, Switzerland, Tel ++41 22 347 62 22

E-Mail: [majorattila@outlook.fr](mailto:majorattila@outlook.fr)

Supplement 2: CINtec® Plus (p16/Ki-67) and High-Risk HPV in patients treated with SAM gel and the control arm

|  |  | | |  | |  | | |  | |  | | |  | |  | |  | |  |  |  |
| --- | --- | --- | --- | --- | --- | --- | --- | --- | --- | --- | --- | --- | --- | --- | --- | --- | --- | --- | --- | --- | --- | --- |
|  |  | | |  | |  | | |  | |  | | |  | |  | |  | |  |  |  |
| **Difference in the CINtec® Plus (p16/Ki-67) after 3 Months (Visit 3)** | | | | | | | | | | | | | | | | |  | |  |  |  |  |
| **ACTIVE ARM** | | | | | | | | **CONTROL ARM** | | | | | | | | |  | |  |  |  |  |
| **Baseline** | | | **3 Months (Visit 3)** | | | | | **Baseline** | | | | **3 Months (Visit 3)** | | | | |  | |  |  |  |  |
|  |  |  | **Missing** | | **Negative** | | **Positive** |  |  |  |  | **Missing** | **Negative** | | **Positive** | |  | |  |  |  |  |
| Negative | | 31 (100.0%) | 1 (3.2%) | | 30 (96.8%) | | 0 (0.0%) | Negative | | 8 (100.0%) | | 0 (0.0%) | 6 (75.0%) | | 2 (25.0%) | |  | |  |  |  |  |
| Positive | | 77 (100.0%) | 5 (6.5%) | | 59 (76.6%) | | 13 (16.9%) | Positive | | 99 (100.0%) | | 1 (1.0%) | 20 (20.2%) | | 78 (78.8%) | |  | |  |  |  |  |
| Missing | | - | - | | - | | - | Missing | | 1 (100.0%) | | 0 (0.0%) | 0 (0.0%) | | 1 (100.0%) | |  | |  |  |  |  |
| Total | | 108 (100.0%) | 6 (5.6%) | | 89 (82.4%) | | 13 (12.0%) | Total | | 108 (100.0%) | | 1 (0.9%) | 26 (24.1%) | | 81 (75.0%) | |  | |  |  |  |  |

The difference in CINtec® Plus test (p16) between arms* (76.6% vs. 20.2%) was significant in favour of the treatment (Fisher´s two-tailed exact test; p<0.001).

|  | |  | |  | | |  | |  | | |  | |  | | |  |  |  |
| --- | --- | --- | --- | --- | --- | --- | --- | --- | --- | --- | --- | --- | --- | --- | --- | --- | --- | --- | --- |
| **Difference in the CINtec® Plus (p16/Ki-67) after 6 Months (Visit 4)** | | | | | | | | | | | | | | | |  |  |  |  |
| **ACTIVE ARM** | | | | | | | | **CONTROL ARM** | | | | | | | |  |  |  |  |
| **Baseline** | | | **6 Months (Visit 4)** | | | | | **Baseline** | | | **6 Months (Visit 4)** | | | | |  |  |  |  |
|  |  |  | **Missing** | | **Negative** | **Positive** | |  |  |  | **Missing** | | **Negative** | | **Positive** |  |  |  |  |
| Negative | 30 (100.0%) | | 2 (6.7%) | | 28 (93.3%) | 0 (0.0%) | | Negative | | 8 (100.0%) | 0 (0.0%) | | 5 (62.5%) | | 3 (37.5%) |  |  |  |  |
| Positive | 72 (100.0%) | | 6 (8.3%) | | 60 (83.3%) | 6 (8.3%) | | Positive | | 98 (100.0%) | 1 (1.0%) | | 21 (21.4%) | | 76 (77.6%) |  |  |  |  |
| Missing | - | | - | | - | - | | Missing | | 1 (100.0%) | 0 (0.0%) | | 0 (0.0%) | | 1 (100.0%) |  |  |  |  |
| Total | 102 (100.0%) | | 8 (7.8%) | | 88 (86.3%) | 6 (5.9%) | | Total | | 107 (100.0%) | 1 (0.9%) | | 26 (24.3%) | | 80 (74.8%) |  |  |  |  |
|  |  | |  | |  |  | |  | |  |  | |  | |  |  |  |  |  |

Of those CINtec® Plus test (p16) positive at baseline, 83.3% and 21.4% patients were screened negative in the active and control arm at the 4^th^ visit. The difference in CINtec Plus® test (p16) result between arms at 6 months was significant (Fisher´s two-tailed exact test; p<0.001).

|  | |  | |  | |  | | |  | |  | |  | |  |  | |
| --- | --- | --- | --- | --- | --- | --- | --- | --- | --- | --- | --- | --- | --- | --- | --- | --- | --- |
|  | |  | |  | |  | | |  | |  | |  | |  |  | |
| **Difference in the High-Risk HPV results after 3 Months (Visit 3)** | | | | | | | | | | | | | |  | | |  |
| **ACTIVE ARM** | | | | | | | **CONTROL ARM** | | | | | | |  | | |  |
| **Baseline** | | | **3 Months (Visit 3)** | | | | **Baseline** | | | **3 Months (Visit 3)** | | | |  | | |  |
|  |  |  | **hr-HPV** | | **No** | |  |  |  | **hr-HPV** | | **No** | |  | | |  |
| hr-HPV | 94 (100.0%) | | 43 (45.7%) | | 51 (54.3%) | | hr-HPV | 85 (100.0%) | | 76 (89.4%) | | 9 (10.6%) | |  | | |  |
| No | 14 (100.0%) | | 0 (0.0%) | | 14 (100.0%) | | No | 23 (100.0%) | | 14 (60.9%) | | 9 (39.1%) | |  | | |  |
| Total | 108 (100.0%) | | 43 (39.8%) | | 65 (60.2%) | | Total | 108 (100.0%) | | 90 (83.3%) | | 18 (16.7%) | |  | | |  |
|  |  | |  | |  | |  |  | |  | |  | |  | | |  |

The high-risk HPV test for the active arm showed that while 94 patients presented with high-risk HPV at the baseline visit, the number of positive tests had decreased at the 3rd visit to 43 patients.

The high-risk HPV test for the control arm showed that while 85 patients presented with high-risk HPV at the baseline visit, the number of positive tests had increased at the 3^rd^ visit to 90 patients.

The difference in high-risk HPV prevalence between arms (54.3% vs. 10.6%) was significant (Fisher´s two-tailed exact test; p<0.001).
